# Multi-Channel Soft Dry Electrodes for Electrocardiography Acquisition in the Ear Region †

**DOI:** 10.3390/s24020420

**Published:** 2024-01-10

**Authors:** Patrick van der Heijden, Camille Gilbert, Samira Jafari, Mattia Alberto Lucchini

**Affiliations:** 1 imec The Netherlands/Holst Centre, 5656 AE Eindhoven, The Netherlands; patrick.vanderheijden@imec.nl; 2 Dätwyler Schweiz AG, 6467 Schattdorf, Switzerlandmattia.lucchini@datwyler.com (M.A.L.)

**Keywords:** electrocardiogram, electrodes, mobile-health, in-ear biosignals, ear-based wearable

## Abstract

In-ear acquisition of physiological signals, such as electromyography (EMG), electrooculography (EOG), electroencephalography (EEG), and electrocardiography (ECG), is a promising approach to mobile health (mHealth) due to its non-invasive and user-friendly nature. By providing a convenient and comfortable means of physiological signal monitoring, in-ear signal acquisition could potentially increase patient compliance and engagement with mHealth applications. The development of reliable and comfortable soft dry in-ear electrode systems could, therefore, have significant implications for both mHealth and human–machine interface (HMI) applications. This research evaluates the quality of the ECG signal obtained with soft dry electrodes inserted in the ear canal. An earplug with six soft dry electrodes distributed around its perimeter was designed for this study, allowing for the analysis of the signal coming from each electrode independently with respect to a common reference placed at different positions on the body of the participants. An analysis of the signals in comparison with a reference signal measured on the upper right chest (RA) and lower left chest (LL) was performed. The results show three typical behaviors for the in-ear electrodes. Some electrodes have a high correlation with the reference signal directly after inserting the earplug, other electrodes need a settling time of typically 1–3 min, and finally, others never have a high correlation. The SoftPulse^TM^ electrodes used in this research have been proven to be perfectly capable of measuring physiological signals, paving the way for their use in mHealth or HMI applications. The use of multiple electrodes distributed in the ear canal has the advantage of allowing a more reliable acquisition by intelligently selecting the signal acquisition locations or allowing a better spatial resolution for certain applications by processing these signals independently.

## 1. Introduction

Mobile health (mHealth) has become an increasingly popular area of research due to its potential to improve access to healthcare services and reduce healthcare costs. In particular, wearable health monitoring devices have been widely adopted as a means of tracking vital signs and health-related behaviors. The in-ear signal acquisition of physiological signals such as ear electrocardiography (ECG) represents a promising addition to this field due to its non-invasive and user-friendly nature. By providing a convenient and comfortable means of physiological signal monitoring, in-ear signal sensing could potentially increase patient compliance and engagement with mHealth applications. This could ultimately lead to more effective disease management and prevention, particularly for cardiovascular diseases, which are the leading cause of death [1] worldwide.

In addition to its potential applications in mobile health, in-ear signal capture has also gained interest for human–machine interface (HMI) applications [2]. In-ear dry electrodes can capture various physiological signals, such as electrocardiography (ECG), electrooculography (EOG), electromyography (EMG), and electroencephalography (EEG) [3,4,5,6,7,8,9,10], which can be used, for example, for sleep stage analysis and to detect and classify cognitive and emotional states. This has the potential to enhance the interaction between humans and machines in various fields, including gaming, virtual reality, and neuroprosthetics [11]. Moreover, the use of in-ear electrodes can provide a discreet and unobtrusive means of signal acquisition, which is particularly important in social or public situations. The development of reliable and comfortable in-ear electrode systems could, therefore, have significant implications for both mHealth and HMI applications. The widespread and ever-increasing use of headphones, hearing aids, and other audio devices makes the ear space a suitable one for the acquisition of physiological signals in real time or as an HMI [12,13,14].

The seamless integration of these functions into existing devices can be achieved through materials similar to those normally used in earpieces, which should meet the challenges of comfort and conformability to the intra- and para-auricular space, as well as the efficient transduction of physiological signals. In this perspective, conductive elastomers offer very interesting properties [15,16]. In particular, SoftPulse^TM^ electrodes (Dätwyler Schweiz AG, Schattdorf, Switzerland) provide a reliable option for acquiring physiological signals over extended periods of time [17].

This publication investigates the use of customized multi-lead SoftPulse^TM^ electrodes for capturing physiological signals, in this case, ECG, from the intra-auricular region. Different electrode placement configurations are studied to reflect various applications that may involve a second electrode at different locations on the body.

In addition, the design of the intra-auricular electrode is such that it can independently acquire the physiological signal from different points inside the ear canal.

## 2. Materials and Methods

### 2.1. Subjects

In total, 5 healthy subjects participated in this study. Subjects were included in this study if they had no known allergic reaction to Ag/AgCl electrodes, healthy skin at the measurement sites, and no impaired hearing or hearing aids. Subjects removed earrings before measurements were started. No skin preparation was performed to reflect typical daily use scenarios. A disposable, non-functional earplug with identical dimensions as the functional earplugs were used prior to the investigation to confirm if the subject was comfortable with wearing the earplug for the duration of the study.

All subjects gave their written informed consent for inclusion before they participated in this study. This study was conducted in accordance with the Declaration of Helsinki, and the protocol was approved by the local ethics board (imec) and Ethics Committee of Máxima MC (METC N22.053).

### 2.2. Materials and Design

The design proposed in this work responds to the desire to measure several physiological signals independently from the ear canal with a single earplug realization. The earplug is designed to collect signals from different sizes and shapes of ear canals while making it comfortable for the user. For this purpose, a customized earplug with conical shape, carrying 6 soft dry electrodes based on Datwyler’s SoftPulse^TM^ technology, was manufactured. The electrodes are cut from sheets of material to form individual electrodes measuring 1 × 1 × 4 mm, arranged longitudinally, and distributed equally around the earplug. The electrodes are carried on a 3D-printed TPU core and are protruding slightly to ensure contact with the participant’s skin. The end of the electrode protruding from the TPU core is clipped to a DIN 1.5 cable that can be connected to the signal capture device.

Each lead can be independently connected to a common reference to yield the differential signal. The electrodes are made of highly electrically conductive elastomer based on Datwyler’s SoftPulse^TM^ technology and coated with a silver–silver chloride-based coating. Other electrode designs have already been presented successfully in the literature for the capture of physiological signals [17,18]. These other designs of soft dry electrodes based on SoftPulse^TM^ technology can be purchased from Datwyler [19]. All components of the earplug have been tested by an independent laboratory to meet the standards of biocompatibility (according to ISO 10993-5 [20], ISO 10993-12 [21], and USP 35-NF30 (87) [22]).

Processing the signals acquired from the ear canal in different ways allows for comparison of the signal quality at different positions and, thus, to study the particularities of this design for signal acquisition. In addition, it allows us to discriminate signals according to their quality during acquisition or processing.

### 2.3. Experimental Setup and Protocol

**Figure 1:**
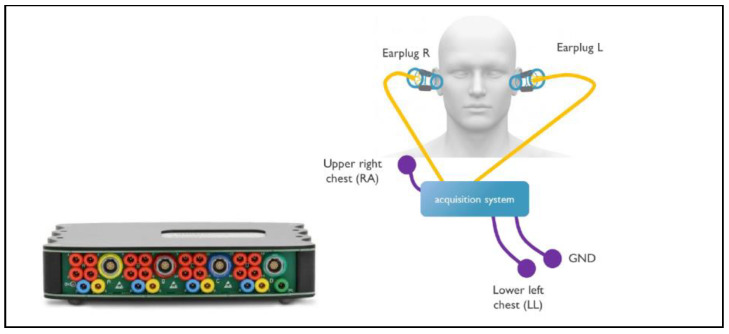
Hardware setup. **Left**: g.USB amp biosignal amplifer. **Right**: earplugs inserted in left and right ear, and electrode placed on chest in standard ECG lead II configuration.

An overview of the experimental setup is shown in Figure 1. The g.USB amp biosignal amplifier (g.tec medical engineering GmbH, Schiedlberg, Austria) was used as a data acquisition platform to capture the biosignals from the 2 earplugs and from 3 electrodes on the chest at a sampling rate of 256 Hz. The frontal ECG lead II was captured with standard off-the-shelf (OTS) electrodes (Kendall 200 Series Foam Electrodes, Cardinal Health, Dublin, OH, USA) between the right arm (RA) and left leg (LL) and 1 grounding electrode and used as the reference for the other measurements. Each earplug captured 6 channels of bioelectric activity in the ear. Through various configurations, ECG could be acquired between the ears and LL, between the 2 ears, and within the ears (see Section 2.4 for details). A headband was used to give additional support to the earplug and the cables attached to it, which minimized changes in earplug position and pressure during data acquisition. This measurement setup was chosen for its versatility and the possibility of selectively configuring the different channels. As the aim of this study was to evaluate the performance of the electrodes only, it was important to eliminate sources of perturbation from the measurement setup by choosing research-grade equipment. The development of capture electronics for in-ear ECG could be the subject of future research.

Subjects were comfortably seated in a chair during the entire recording. After placing the earplugs, gel electrodes, and headband, the protocol steps were as follows:

1. Measurement between each ear and LL;
2. Measurement between ears;
3. Measurement within ear.

**Figure 2:**
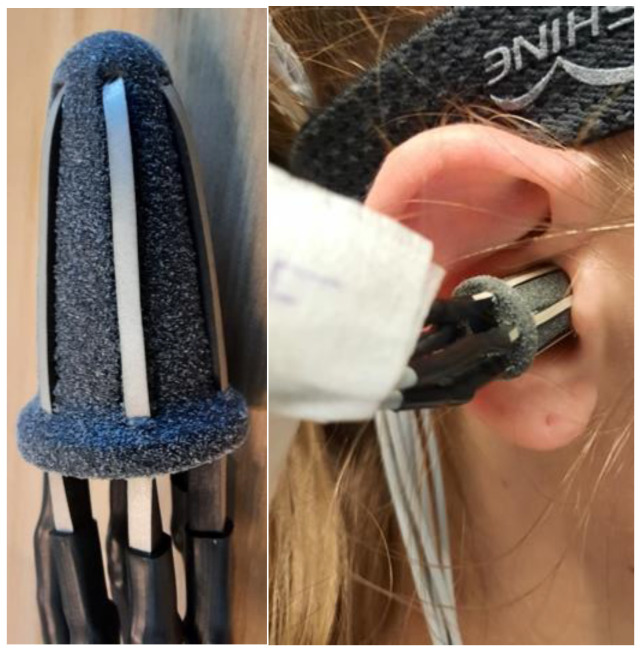
**Left**: Earplug. **Right**: Earplug inserted in right ear of 1 of the volunteers.

The earplugs were placed in the ear canal in a random rotation as shown in Figure 2. Hence, the position of the electrodes was also random and not controlled during the data collection. However, the position of the electrodes was noted down, which allowed for rotation correction during post-processing. After concluding data collection, the earplugs and electrodes were removed, and subjects were asked about their comfort regarding the usage of the earplugs.

### 2.4. Data Acquisition

**Figure 3:**
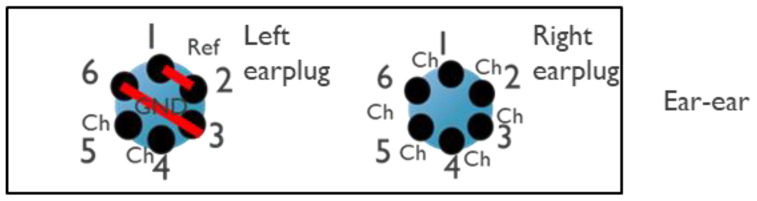
Shortening of individual electrodes during the ear-to-ear measurement. The red lines in the left part of the picture show the electrodes that have been shortened in the experiment. In particular, electrodes 1 and 2 and electrodes 3 and 6 are shortened for the measurement between the ears.

**Figure 4:**
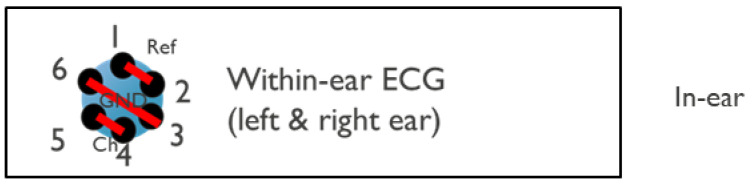
Shortening of individual electrodes during the measurement within the ears. The red lines in the left part of the picture show the electrodes that have been shortened in the experiment. In particular, electrodes 1 and 2, 3 and 6, and 4 and 5 are shortened for the measurement within the ears.

Data were acquired using the g.USBamp biosignal amplifier. The hardware setup during the protocol steps is outlined below:

1. Measurement between each ear and LL (12 channels)
  - The 6 electrodes of each earplug (12 in total) were connected to the ‘channel’ inputs of the acquisition system. The differential biopotentials between each of these 12 electrodes and a gel electrode in LL were measured.
2. Measurement between ears (6 channels), presented in Figure 3
  - Electrodes 1 and 2 and electrodes 3 and 6 of the left earplug were shortened and connected to the ‘ref’ and ‘gnd’ connection of the data acquisition system, respectively. Electrodes 4 and 5 were kept floating.
  - The right earplug electrodes were connected to the ‘channel’ inputs of the data acquisition system.
3. Measurement within ear (2 channels: 1 from left ear, 1 from right ear), presented in Figure 4
  - For each earplug, electrodes 1 and 2, electrodes 3 and 6, and electrodes 4 and 5 were shortened and connected to the ‘ref’, ‘gnd’, and ‘channel’ inputs of the acquisition system, respectively.

During each of the 3 protocol steps, the frontal ECG lead II was captured with standard off-the-shelf (OTS) electrodes (Kendall 200 Series Foam Electrodes) between the right arm (RA) and left leg (LL) and 1 grounding electrode. This ECG served as a reference signal. No online filtering was applied during data acquisition.

### 2.5. Data Processing and Analysis

Each measured channel was filtered offline with a 2nd order Butterworth low-pass (fc = 30 Hz) and high-pass filter (fc = 0.3 Hz) in forward and backward directions to minimize phase distortion (Matlab 2021a). ECG beats were detected from the reference ECG using a beat detection algorithm. A template ECG was constructed for each channel by time synchronous averaging, in which waveforms obtained by extracting the ECG epochs around the detected beats are averaged in windows of 16 s with 8 s overlap. Correlation between the earplug measurement and reference signal was computed. A moving average of 3 correlation samples is applied to slightly smooth the correlation plot and improve readability.

## 3. Results

The following section describes the results of the individual steps from the protocol.

### 3.1. Measurement between Each Ear and LL

**Figure 5:**
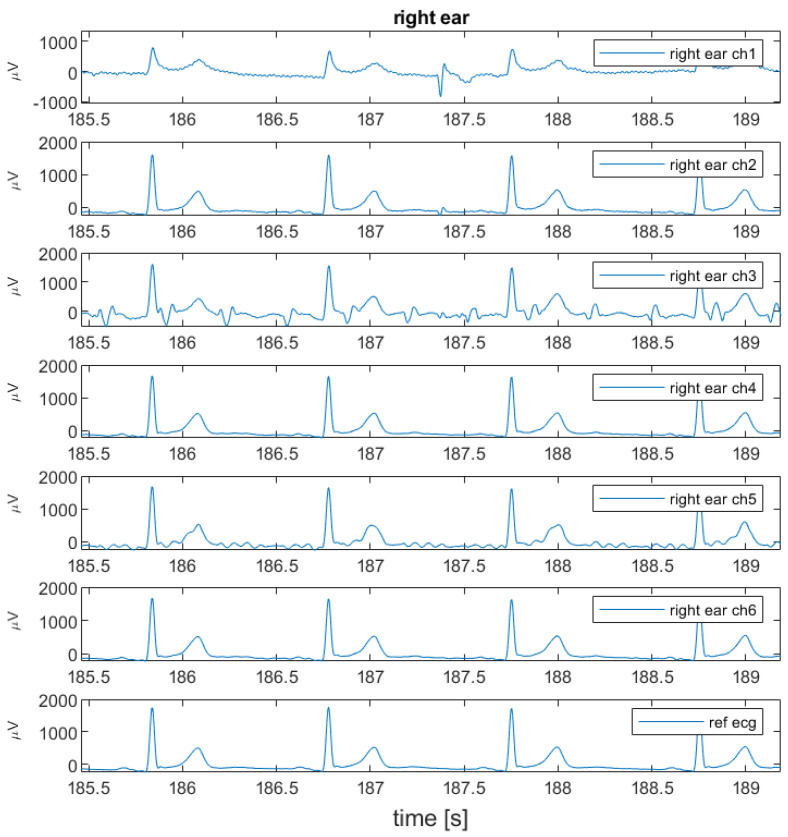
Example of raw data measured between right ear and LL. From top to bottom, the 6 measurement channels and the reference signal (lead II) are shown.

**Figure 6:**
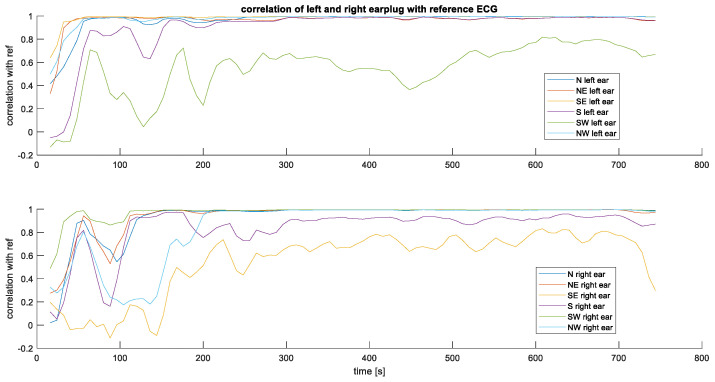
Correlation plot of the left (**top plot**) and right (**bottom plot**) earplug with the reference signal. N = north (top-most electrode), E = East, S = South, W = West.

**Figure 7:**
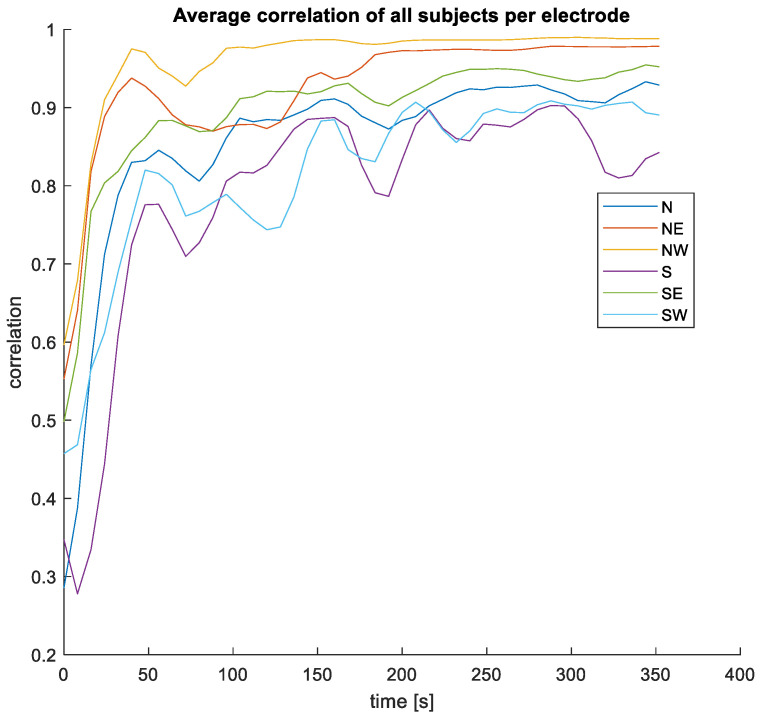
Averaged correlation of all subjects as function of time and position in the ear.

Figure 5 shows an example of the raw unfiltered measurements taken in the first configuration. Figure 6 shows the correlation over time between the earplug measurements and the reference signal. In Figure 6, the earplug channels are remapped according to their position in the ear. As can be seen in the example, the correlation is high for most of the channels, especially after allowing a settling time of a few minutes. From all correlation and raw signal plots, three typical behaviors are observed: (1) some electrodes have a high correlation with the reference signal directly after inserting the earplug, (2) other electrodes need a settling time of typically 1–3 min, and (3) others never have a high correlation. For most measurements, one or two electrodes at the bottom position (South) show bad correlation values. Average correlation values are depicted in Figure 7. The correlation values at the end of the recording are 0.93, 0.98, and 0.99 for the topmost electrodes (N, NE, and NW electrodes, respectively) and 0.84, 0.95, and 0.89 for the bottommost electrodes (S, SE, SW electrodes, respectively). After examining the disparities between the left and right ear, no notable differences were observed in terms of SNR or correlation values.

### 3.2. Ear-to-Ear Measurement

**Figure 8:**
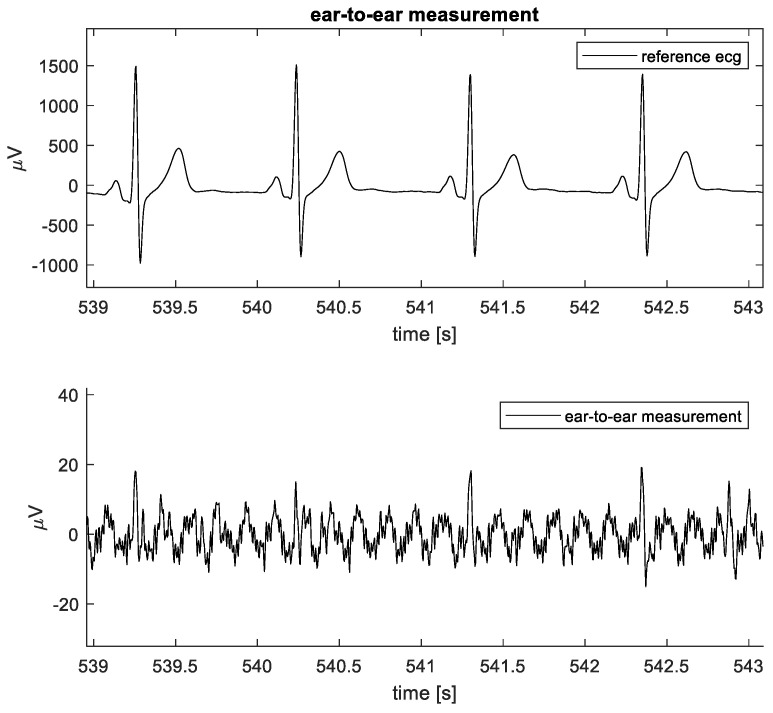
Example of ear-to-ear ECG data captured for 1 subject. Reference ECG (**top plot**) and ear-to-ear measurement (**bottom plot**).

**Figure 9:**
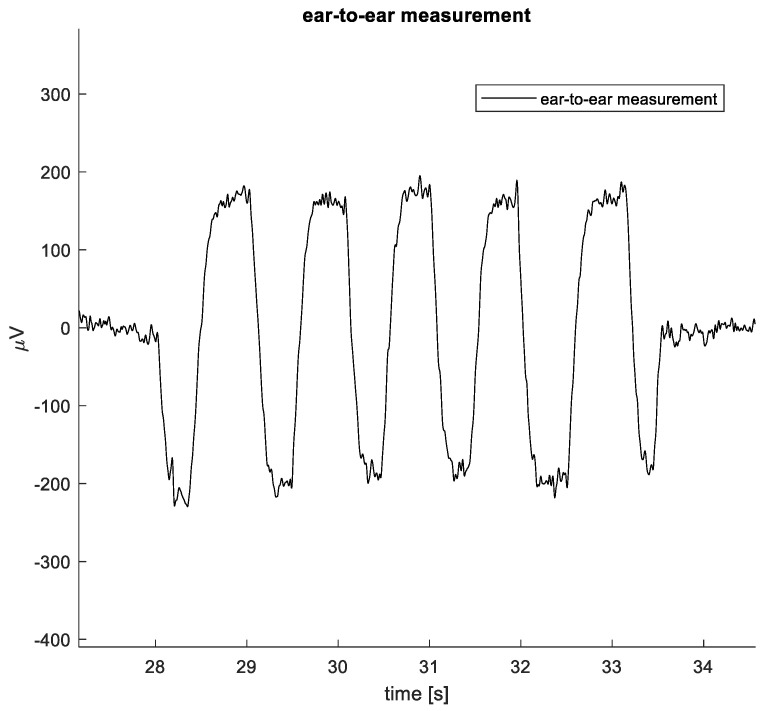
Ear-to-ear measurement showing horizontal eye movements.

The QRS amplitude of the ECG measured between the left and right ear was approximately 25 µV for one subject and 50 µV for two subjects. For the other two subjects, no QRS peak was detected. An example of the ECG measured between the ears is shown in Figure 8’s bottom graph. Overall, the noise level of the setup is low enough to allow us to notice distinct ECG beats, although the noise of various origins can be present. The example clearly shows the distinct ECG beats with 6 Hz sinusoidal noise present, most likely an aliased component. The eye activity (EOG) can also be picked up during ear-to-ear measurements. Horizontal eye movements created a signal between the ears with an amplitude of approximately 400 µV pp (see also Figure 9). Here, the subject moved the eyes as far as possible from left to right several times.

### 3.3. Within Ear ECG

**Figure 10:**
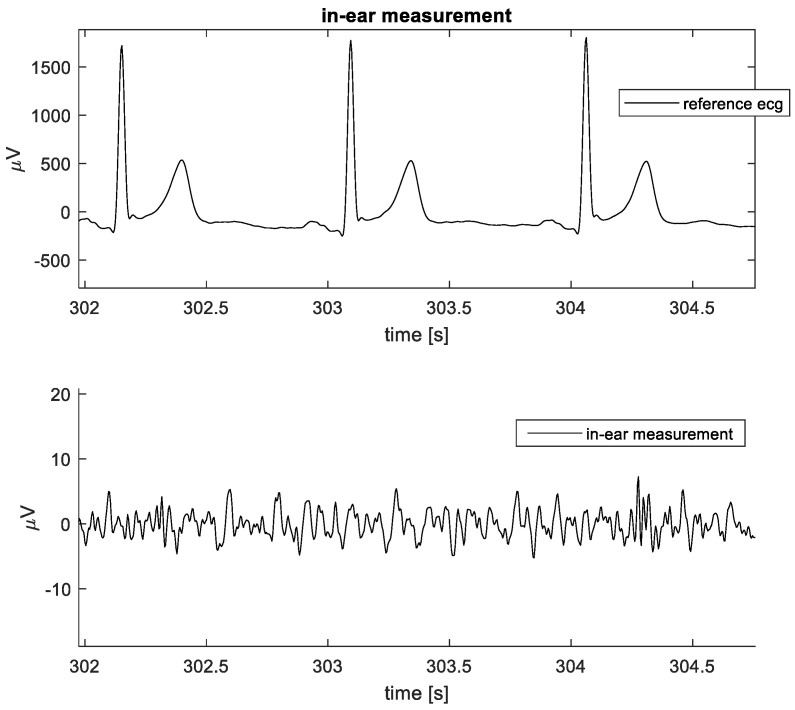
Example of data captured during the in-ear data collection. **Top**: Reference lead II ECG. **Bottom**: In-ear data.

No ECG could be visually observed from any of the measurements within the ears with the setup. Peak noise levels were around ±5 µV, as illustrated in Figure 10.

## 4. Discussion

Below, we discuss several aspects that play an important role in in-ear bio signal acquisition using soft dry electrodes.

### 4.1. Suitability of the Ear for Bio Signal Data Acquisition

**Figure 11:**
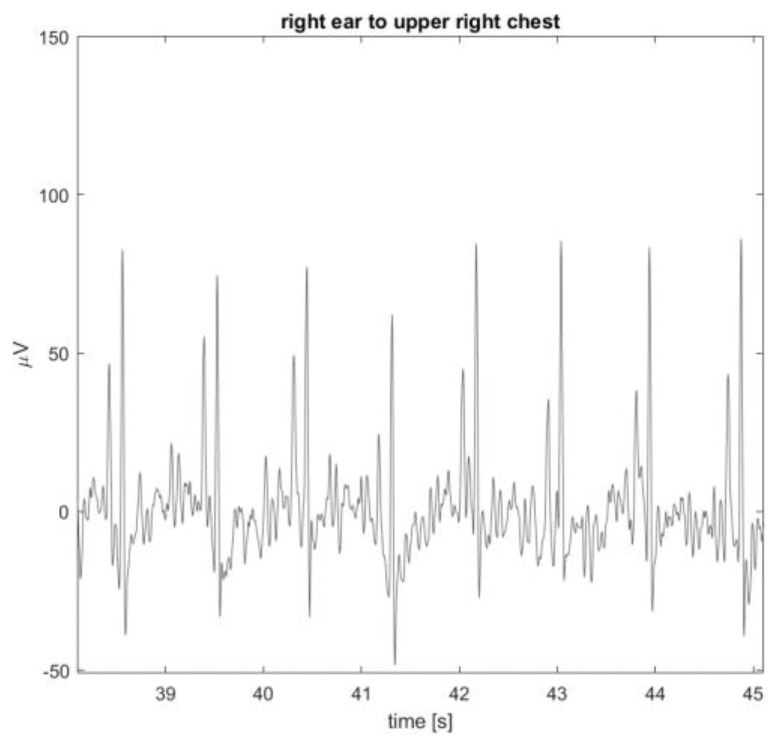
Example of data captured between ear and upper right chest on top of collar bone.

The ears seem to be a suitable location for capturing biosignals, as demonstrated in this work, where we use multi-lead SoftPulse^TM^ earplugs with six integrated leads, as well as from the literature [3,4,5,6,7,8,9,10,18]. When referenced to another measurement point on or below the neck, it could be possible to capture a high-quality bipolar ECG between the ear and the other measurement point. For example, the earplug cable could be lying on the neck and be made conductive, allowing for a bipolar measurement between the ear and neck. In Figure 11, a data example for this scenario is shown, depicting the ECG signal measured between the right ear (earplug) and upper right chest on top of the collar bone. QRS peak amplitude increased with inter-electrode distance, in this case, to approximately 110 µV pp.

This approach paves the way for unobtrusive, long-term, and low-power ECG acquisition. Between the ears, the horizontal EOG could be acquired together with a small-amplitude ECG, although the latter was with large inter-subject variability. The ECG measured between the ears is smaller in amplitude and has a lower SNR, which is likely due to the distance to the heart and the volume through which the ECG wavefront needs to propagate. Regarding the within-ear ECG, satisfactory low noise levels are observed in our hardware setup, which should have been enough to capture an ECG with an amplitude around 10–15 µV pp reported in the literature [23]. However, this was not the case. To further investigate this, future studies should control accurately the positioning of the bipolar in-ear measurements to gain a deeper understanding of the possibilities in this case.

### 4.2. Stabilization Time

**Figure 12:**
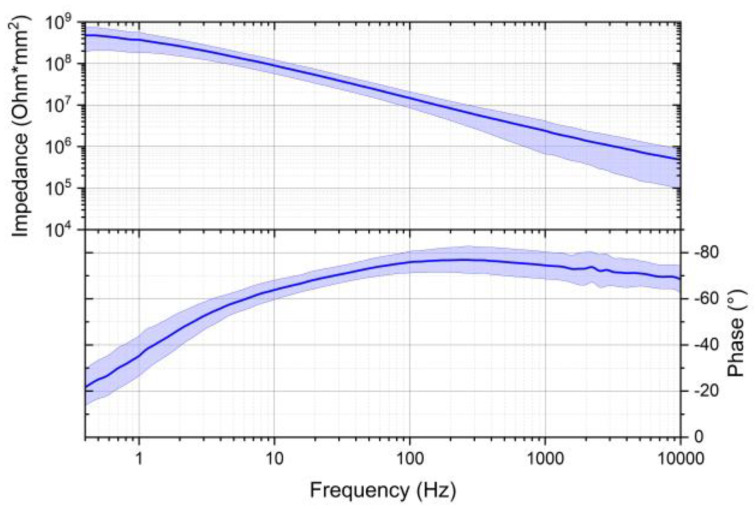
Impedance and phase shift of the SoftPulse^TM^ soft dry electrodes coated with silver–silver chloride-based coating.

**Figure 13:**
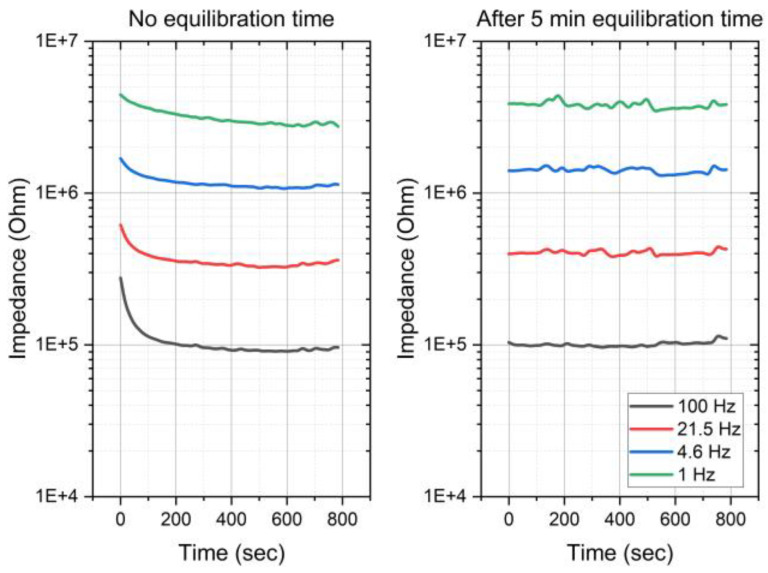
Equilibration time for impedance stabilization of SoftPulse^TM^ soft dry electrodes coated with silver–silver chloride-based coating.

Within this study, stabilization time is defined as the time needed to consistently reach a correlation between the earplug signal and a reference ECG above 0.98. Some electrodes showed a high correlation with the reference ECG directly after inserting the earplug, other channels need a settling time of typically 1–3 min, and others never have a high correlation. The impedance of the electrodes was assessed utilizing the three-electrodes configuration [17], and the results are illustrated in Figure 12. Figure 13 provides a visual representation of the established equilibration time necessary to achieve a stable impedance with electrodes of this nature. The recorded impedance measurements align with observed settling times in the majority of the conducted measurements via correlation with the measured signals with the reference ECG.

For most measurements, one or two of the bottom (South) channels show bad correlation values. This could be attributed to differences in the electrode contact pressure exerted within the ear. Both the angle of earplug insertion and tension exerted by the cables on the earplug contributed to consistent variations within this experimental setup, where the bottom electrodes showed the lowest performance. Adding electrode–tissue impedance measurements per channel could provide additional insights into settling time and contact pressure in future research.

### 4.3. Differences between the Left and Right Ear

When measuring the bipolar ECG between the lower left chest (LL) and the left or right ear, the results show that both ears are equally suitable for collecting ECG in that case. No significant differences in SNR or correlation values were observed in terms of stabilization time or overall signal quality. This proves the suitability of both ears as a location for bio signal acquisition.

### 4.4. Comfort of In-Ear Solutions

After concluding the experiments, the subjects were asked to rate how they felt about the comfort of the earplug. The comfort level was consistently rated as acceptable without exception. No pain or irritation was reported during and after the experiments. This shows the suitability of the form factor and measurement location. Important factors that contribute to the comfort of measurements within the ear are the shape and dimension of the earplug, as well as the materials used.

## 5. Conclusions

This research evaluates the quality of the ECG signal obtained with customized multi-lead SoftPulse^TM^ electrodes inserted in the ear canal. An earplug with six leads distributed around its perimeter was designed for this study. We conclude that the soft dry electrodes used in this research have proven to be perfectly capable of measuring ECG signals, paving the way for their use in mHealth or the HMI applications, particularly via the in-the-ear devices used on a daily basis, such as hearing aids or portable headphones. The use of several leads distributed in the ear canal has the advantage of allowing a more reliable acquisition by intelligently selecting the best locations or allowing a better spatial resolution for certain applications by processing these signals independently.

## Data Availability

The datasets presented in this article are not readily available because of data privacy clauses. Requests to access the datasets should be directed to camille.gilbert@datwyler.com.
